# Gut Ischemia Reperfusion Injury Induces Lung Inflammation via Mesenteric Lymph-Mediated Neutrophil Activation

**DOI:** 10.3389/fimmu.2020.586685

**Published:** 2020-09-11

**Authors:** Yonggang Ma, Taylor Zabell, Alexandra Creasy, Xiaoyuan Yang, Victor Chatterjee, Nuria Villalba, Erik B. Kistler, Mack H. Wu, Sarah Y. Yuan

**Affiliations:** ^1^Department of Molecular Pharmacology and Physiology, University of South Florida Morsani College of Medicine, Tampa, FL, United States; ^2^Department of Anesthesiology and Critical Care, University of California, San Diego, Veterans Affairs San Diego Healthcare System, San Diego, CA, United States; ^3^Department of Surgery, University of South Florida Morsani College of Medicine, Tampa, FL, United States; ^4^James A. Haley Veterans’ Hospital, Tampa, FL, United States

**Keywords:** acute lung injury, inflammation, intestinal ischemia/reperfusion, mesenteric lymph, neutrophils, permeability

## Abstract

Gut ischemia/reperfusion (I/R) injury is a common clinical problem associated with significant mortality and morbidities that result from systemic inflammation and remote organ dysfunction, typically acute lung injury. The mechanisms underlying the dissemination of gut-derived harmful mediators into the circulation are poorly understood. The objective of our study was to determine the role of mesenteric lymphatic circulation in the systemic and pulmonary inflammatory response to gut I/R. Using a murine intestinal I/R model, we evaluated whether and how blocking mesenteric lymph flow affects the inflammatory response in local tissues (gut) and remote organs (lungs). We further explored the mechanisms of post-I/R lymph-induced systemic inflammation by examining neutrophil activity and interaction with endothelial cells *in vitro*. Mice subjected to intestinal I/R displayed a significant inflammatory response in local tissues, evidenced by neutrophil infiltration into mucosal areas, as well as lung inflammation, evidenced by increased myeloperoxidase levels, neutrophil infiltration, and elevated microvascular permeability in the lungs. Mesenteric lymph duct ligation (MLDL) had no effect on gut injury *per se*, but effectively attenuated lung injury following gut I/R. Cell experiments showed that lymph fluid from post-I/R animals, but not pre-I/R, increased neutrophil surface CD11b expression and their ability to migrate across vascular endothelial monolayers. Moreover, post-I/R lymph upregulated neutrophil expression of pro-inflammatory cytokines and chemokines, which was mediated by a mechanism involving nuclear factor (NF)-κB signaling. Consistently, gut I/R activated NF-κB in lung neutrophils, which was alleviated by MLDL. In conclusion, all these data indicate that mesenteric lymph circulation contributes to neutrophil activation and lung inflammation following gut I/R injury partly through activating NF-κB.

## Introduction

The gut plays a central role in the development of systemic inflammation and multiple organ dysfunction, including acute lung injury (ALI), a life-threatening condition commonly seen in critically ill patients such as those with sepsis, major trauma, shock, burn, and severe infection. The gut origin of infection or inflammation serves as not only an important cause but also effector of systemic injury. For example, trauma or sepsis is often associated with intestinal ischemia/reperfusion (I/R) injury, which results in gut barrier dysfunction and translocation of bacteria or injurious factors to the systemic circulation and remote tissues, further exacerbating organ dysfunction (1). Neutrophil activation and infiltration into the lungs are a hallmark of ALI, where activated neutrophils cause tissue damage through multiple mechanisms, including secreting pro-inflammatory mediators (2, 3). However, the underlying mechanisms by which gut I/R leads to lung neutrophil infiltration and activation are incompletely understood.

The mesenteric lymphatic system is crucial in maintaining circulatory homeostasis. It drains gut interstitial fluid and proteins to prevent tissue edema, transports antigens and immune cells for immune surveillance, and absorbs dietary lipids and fat-soluble vitamins to supply the whole body (4). Interstitial fluid drains into lymphatic capillaries (lacteals) forming lymph, and lymph flows via a network of lymphatic capillaries, collecting vessels, nodes, and duct into the blood circulation through subclavian vein (5). Accumulating evidence has implicated the mesenteric lymph in ALI following hemorrhagic shock (6), burn (7), endotoxemia (8), and severe intraperitoneal infection (9). As all these studies were carried out in rodent models of injury that induce global homeostatic disturbance not originated from the gut, they are challenged by the difficulty in interpretation of the gut as the motor of critical illness. While an *in vitro* study shows that post-injury lymph can prime neutrophils for enhanced respiratory burst (10), the identity of lymph injurious factors and the signaling mechanisms underlying their effects remain largely elusive. This study is designed to provide molecular insights into how post-I/R lymph contributes to neutrophil activation and lung inflammation. In order to distinguish the gut origin, we used a mouse model of intestinal I/R and tested the hypothesis that mesenteric lymphatics serve as an important conduit of gut-lung crosstalk in inflammation.

## Materials and Methods

### Animals

C57BL/6J mice (3–6 month old, male) purchased from Jackson Laboratory were used in this study. Male Sprague-Dawley rats weigh >200 g were purchased from Harlan Laboratories. All animals were acclimated for at least 1 week before use. Animals were housed in a facility with a 12/12 h light/dark cycle and had free access to water and standard rodent chow. All animal procedures were approved by the Institutional Animal Care and Use Committee at the University of South Florida and were conducted in accordance with the Guide for the Care and Use of Laboratory Animals (Eighth edition revised in 2011).

### Intestinal I/R and Mesenteric Lymph Duct Ligation (MLDL)

The mouse was placed on its right side under anesthesia with isoflurane. The abdomen was opened extending about 1.5 cm laterally from the xyphoid, and the superior mesenteric artery was occluded below the celiac trunk with an arterial clamp. After 30 min, the clamp was removed to reperfuse the intestine (11). In some animals, the white-colored mesenteric lymph duct was isolated and ligated with 7-0 suture before inducing intestinal ischemia. The abdomen was closed with 4-0 suture, followed by resuscitation with 1 mL of lactated ringer’s solution. Sham controls received the same procedure with the exception of artery occlusion.

### Perfusion and Tissue Collection

At 4 h after intestinal I/R, mice were anesthetized. The lungs were perfused with lactated ringer’s solution from right ventricle. Right lung lobes were collected for protein extraction. The left lobe was perfused with 10% formalin and collected for histology. The small intestine of jejunum and ileum was collected for protein and histology use (fixed with 10% formalin).

### Immunohistochemical Staining

Paraffin-embedded sections (5 μm) were deparaffinized in xylene and rehydrated through graded ethanol. Heat mediated antigen retrieval was conducted using IHC-Tek Epitope Retrieval Solution (IW-1100). Neutrophils were stained using an antibody specific for neutrophils (Cederlane, CL8993AP). Immunostaining was performed using the Vectastain Elite ABC Kit (Vector, PK-6104). DAB substrate kit (Vector, SK-4100) was used to visualize positive staining, with eosin as a counterstain. Quantification was expressed as numbers of positive cells per 40× high magnification field or as the percentage of positively stained area to total area. Six to ten random scans per section were analyzed and averaged (12).

### Immunoblotting

Tissues or cells were homogenized in 1× RIPA lysis buffer (Millipore, 20-188) with proteinase and phosphatase inhibitors (Roche, 04693124001, 04906845001), and the supernatant was collected after centrifugation at 14,000 × *g* for 10 min. Protein quantification was performed using BCA protein assay (Pierce, 23227). Total protein for all samples were separated on 4–20% Criterion^TM^ XT Bis-Tris gels (Bio-Rad), transferred to nitrocellulose membrane (Bio-Rad), and stained with Revert^TM^ 700 Total Protein Stain Kit (LI-COR, 926-11016) to verify protein concentration and loading accuracy. After blocking with Odyssey blocking buffer (LI-COR, 927-70001), the membrane was incubated with a goat anti mouse/human myeloperoxidase (MPO) antibody (R&D, AF3667), a rabbit anti-mouse phospho (p)-p65 nuclear factor (NF)-κB (CST, 3033S), or a rabbit anti-mouse p65 NF-κB (CST, 8242S) overnight at 4°C, followed by incubation with a donkey anti-goat or anti-rabbit 680RD secondary antibody (LI-COR, 925-68074 or 926-68073) for 45 min at room temperature. The signal was measured at the wavelength of 700 nm with the LI-COR imaging system (Odyssey CLx). The signal of total protein was used as the internal loading control for each lane, and data were quantified as the ratio of MPO to total protein signal (12). The ratio of p-p65 to p65 was quantified to represent NF-κB activation.

### Quantitative Real Time PCR

Total RNA was extracted using TRIzol^®^ Reagent (Invitrogen, 15596). RNA concentration was measured using the NanoDrop ND-2000 Spectrophotometer (Thermo Fisher Scientific). Reverse transcription of RNA was performed using High Capacity RNA-to-cDNA Kit (Thermo Fisher Scientific, 4387406), and gene expression was measured using Taqman Gene Expression Master Mix (Thermo Fisher Scientific, 4369016) plus individual primers for *Il1b*, *Il6*, *Il12a*, *Tnf*, *Ccl3*, *Ccl5*, *Cxcl1*, *Cxcl2*, and *Cxcl5*. The gene levels were normalized to the reference gene *Gapdh* and expressed as fold change to control groups. MIQE guidelines were followed for all the PCR experiments and analysis (13).

### Lung Microvascular Permeability Assays

After anesthesia, mice were intravenously injected with 1% Evans blue dye (100 mg/kg). After 30 min, the lungs were perfused with lactated ringer’s solution to remove intravascular Evans blue dye (14). The entire right lobes were homogenized in 0.5 mL of PBS, incubated with 1 mL of formamide (Fisher Scientific, BP227-500) at 60°C for 18 h to extract Evans blue, and centrifuged at 5,000 *g* for 30 min to collect the supernatant. The concentration of Evans blue was detected by measuring absorption at the wavelength of 620 nm (15). The left lobe was visualized at the wavelength of 700 nm with the LI-COR imaging system.

### Mesenteric Lymph Collection

Under anesthesia with isoflurane, a ∼4 cm incision was made from midline to the right flank of the rat abdomen. The mesenteric lymph duct, paralleled to the superior mesenteric artery, was bluntly dissected. A small hole in the lymph duct was made with microscissors, and the PE10 tube filled with heparinized saline was inserted into the lymph duct (16). The pre-I/R normal lymph was collected to sterile tubes. The superior mesenteric artery was occluded for 45 min, and then reperfused. Post-I/R lymph at 0–2 h after intestinal I/R was collected. The lymph was centrifuged at 400 *g* for 10 min to remove cells and stored at −80°C for future use.

### Endothelial Barrier Function *in vitro*

Endothelial barrier function was evaluated using an electric cell-substrate impedance sensing system (Applied Biophysics), as described previously (14, 15). Human umbilical vein endothelial cells were seeded on 8W10E + electrode chambers and cultured to confluence. Pre-I/R lymph (10%), post-I/R lymph (10%), thrombin (10 U/mL, Sigma, T7513), or vehicle controls was added to the chamber. Real time changes in transendothelial electrical resistance (TEER) was recorded and analyzed by normalizing to its own baseline.

### Mouse Bone Marrow Neutrophil Isolation

Mouse bone marrow neutrophils were isolated as described by Swamydas and colleagues (17). Briefly, femurs and tibias were flushed with RPMI 1640 media supplemented with 10% FBS and 2 mM EDTA. The red blood cells were lysed with hypotonic 0.2% NaCl, followed by 1.6% NaCl to restore to normal osmolarity. Neutrophils were then separated by density gradient centrifugation using the Histopaque 1119 and 1077 (Sigma). The isolated neutrophils were utilized for *in vitro* studies.

### Flow Cytometry

Heparin anti-coagulated whole blood was collected from normal mice. The blood samples were stimulated with pre-I/R or post-I/R lymph for 100 min and stained with FITC CD45 (Biolegend, 103108), APC CD11b (Biolegend, 101212), and PE Ly6G (Biolegend, 127608) antibodies for 20 min. After lysing red blood cells (Miltenyi Biotec, 130-094-183), the samples were run on a BD FACSCanto II flow cytometer. Neutrophils were identified as CD45^+^CD11b^+^ Ly6G^+^ cells. The mean fluorescent intensity (MFI) of CD11b on neutrophils was measured. In another set of experiments, bone marrow derived neutrophils isolated from normal mice were incubated with pre-I/R or post-I/R lymph and stained with CD11b antibody. CD11b expression was measured as described above. The lymph concentration used (5 and 10%) was based on the ratio of mesenteric lymph volume produced in the first 2 h after injury to blood volume, as reported by others (18, 19).

### Neutrophil Transmigration Assay

Mouse lung microvascular endothelial cells were cultured to confluence on 24-well transwell inserts (Costar, 3.0 μm). Neutrophils pretreated with vehicle, pre-I/R lymph, or post-I/R lymph for 2 h were added to the top chamber and incubated for 2 h at 37°C with recombinant CXCL1 (100 ng/mL, R&D, 453-KC/CF) in the bottom chamber as the chemoattractant. The number of neutrophils transmigrated into the bottom chamber was counted. In a separate set of experiments, neutrophils were stained with Cellmask green plasma membrane (Invitrogen, C37608), and transmigrated neutrophils were visualized using Olympus FV1200 laser scanning confocal microscope (14).

### Neutrophil Stimulation

For PCR experiments, neutrophils were stimulated for 4 h, and RNA was extracted. For experiments with blocking reagents, the neutrophils were incubated with inhibitors or antagonists for 1 h, and then stimulated with lymph for additional 4 h. Toll like receptor (TLR) 4 inhibitor (5083360001), diamine oxidase (DAO) inhibitor (109266), and NF-κB inhibitor (481406) were purchased from Millipore Sigma. Complement C3a receptor antagonist (C3aRa) SB 290157 (6860) and C5a receptor antagonist (C5aRa) PMX 53 (5473) were purchased from Tocris Bioscience.

### Neutrophil Respiratory Burst Assay

Neutrophil respiratory burst assay was performed according to manufacturer’s instruction (Abcam, ab236210). Briefly, isolated bone marrow neutrophils were incubated with dihydrorho-damine 123 assay reagent for 15 min at 37°C, followed by stimulation with vehicle, 10% pre-I/R lymph, 10% post-I/R lymph, or phorbol myristate acetate (PMA, 200 nM) for 45 min at 37°C. The dihydrorhodamine 123, a cell permeable, non-fluorescent dye, is converted to the fluorescent compound rhodamine 123 by reactive species produced by activated neutrophils. The MFI at 530 nm was measured using a BD FACSCanto II flow cytometer.

### Extracellular DNA Measurement

Isolated bone marrow neutrophils were stained with SYTOX Green nucleic acid stain (5 μM, ThermoFisher, S7020) and then stimulated with vehicle, 10% pre-I/R lymph, 10% post-I/R lymph, or calcium ionophore (5 μM, Sigma, A23187) for 4 h at 37°C. The fluorescence intensity (emission: 527 nm) was measured at 0, 1, 2, 3, and 4 h after stimulation.

### Neutrophil Degranulation Assay

Isolated bone marrow neutrophils were stimulated with vehicle, 10% pre-I/R lymph, 10% post-I/R lymph, or PMA (200 nM) for 30 min in RPMI1640 media with 1% FBS. The supernatant was collected and run for immunoblotting to measure MPO levels. The degranulation was calculated as subtraction of the intensity of lymph endogenous MPO by the intensity of supernatant MPO and expressed as fold changes to controls.

### Detection of Bacteria RNA in Lymph

Pre-I/R and post-I/R lymph were plated onto tryptic soy agar to test for bacterial growth. Concentrations of plated lymph were 100, 50, 20, and 10%, diluted with sterile tryptic soy broth. Plates were then incubated for 24 h at 37°C. Bacterial DNA was amplified via PCR utilizing the 16S rRNA primers 27F and 1492R (20).

### Cytokine Array in the Lymph

Eighty nine cytokine levels in the lymph were measured using a rat XL cytokine array kit (R&D, ARY030). Briefly, the membrane was incubated with 200 μL of pre-I/R or post-I/R lymph and reagents according to the manufacture instruction. IRDye 800CW streptavidin (LI-COR, 926-32230) was applied on the membrane, and the intensity of signal was measured using the LI-COR imaging system and normalized to references.

### Immunofluorescence

Bone marrow neutrophils were plated on coverslips in a 24-well plate for 2 h, followed by stimulation with 10% pre-I/R lymph, 10% post-I/R lymph, or media for 1 h. After fixation, permeabilization, and blocking, the cells were stained with p-p65 or p65 antibody overnight at 4°C, followed by incubation with an Alexa Fluor 488 donkey anti-rabbit secondary antibody (Invitrogen, A21206) for 1 h at room temperature. The nuclei were stained with DAPI, and images were taken using a Leica SP8 confocal microscope.

### Lung Neutrophil Isolation

At 4 h after intestinal I/R, the lungs were perfused and dissected. Lung tissue was minced into small pieces and digested with Liberase TL (350 U/mL, Roche, 054010200001) and DNase I (5 U/mL, Roche, 41407200). The cell suspension was applied over a 40 μm cell strainer to remove non-dissociated clumps, and red blood cells were lysed with red blood cell lysis solution (Miltenyi Biotec, 130-094-183). The single cell suspension was incubated with anti-Ly6G MicroBeads UltraPure (Miltenyi Biotec, 130-120-337) and applied over a magnetic MS column (Miltenyi Biotec, 130-042-201). Ly6G^+^ neutrophils adhered to columns were collected (21).

### Statistical Analysis

Data are presented as mean ± SEM. Multiple group comparisons were performed using ordinary one-way ANOVA and Tukey’s multiple comparisons test when the Bartlett’s variation test was passed or using the non-parametric Kruskal-Wallis test and Dunn’s multiple comparisons test when the Bartlett’s variation test did not pass. Two group comparisons were analyzed by parametric test when data conform to Gassian distribution or non-parametric test when data do not conform to Gassian distribution. A value of *p* < 0.05 was considered statistically significant.

## Results

### Gut I/R-Induced Neutrophil Infiltration in Local Tissues Is Not Affected by Blocking Mesenteric Lymph Flow

**FIGURE 1:**
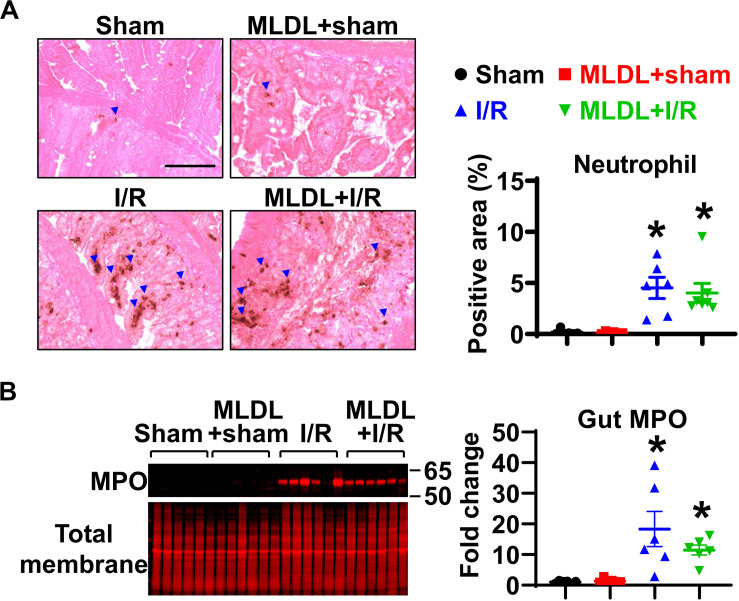
Gut I/R-induced neutrophil infiltration in local tissues is not affected by blocking mesenteric lymph flow. **(A)** Immunohistochemical staining reveals increased gut neutrophil numbers in I/R, which is not affected by mesenteric lymph duction ligation (MLDL). The blue arrowheads indicate neutrophils (brown dots). Scale bar, 100 μm. **(B)** Myeloperoxidase (MPO) expression in the small intestine is elevated after I/R, which is not altered by MLDL. *n* = 6–7/group. *p < 0.05 vs. sham or MLDL + sham. One-way ANOVA was used.

To determine whether MLDL affects gut neutrophil infiltration after I/R, we measured neutrophil numbers in the small intestine. Immunohistochemical staining of neutrophils demonstrated that gut I/R dramatically induced neutrophil infiltration into the mucosa (Figure 1A). MLDL did not impact gut neutrophil levels, regardless of I/R status (Figure 1A). Consistently, the expression of gut MPO, a marker of neutrophils, significantly increased in both I/R and MLDL + I/R animals, compared to sham controls (Figure 1B). However, MLDL did not alter gut MPO expression.

### MLDL Attenuates Intestinal I/R-Induced Neutrophil Infiltration and Lung Microvascular Permeability

**FIGURE 2:**
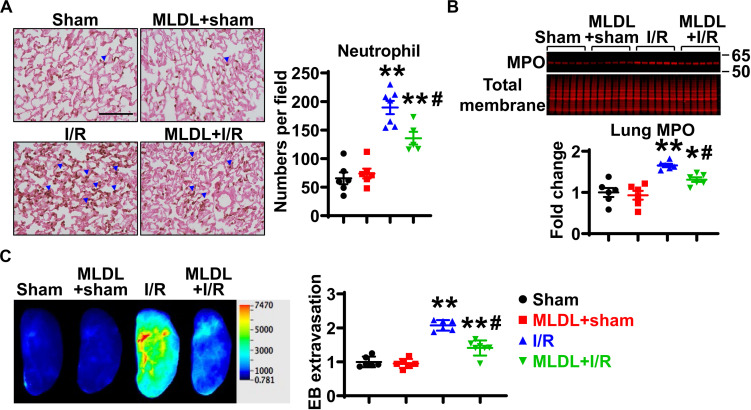
MLDL attenuates intestinal I/R-induced Lung neutrophil infiltration and microvascular permeability. **(A)** Intestinal I/R causes neutrophil recruitment into the lung, which is attenuated by MLDL. The blue arrowheads indicate neutrophils (brown dots). Scale bar, 100 μm. **(B)** Intestinal I/R elevates lung MPO expression, an effect ameliorated by MLDL. **(C)** Lung microvascular permeability (Evans blue extravasation) is increased after gut I/R, which is alleviated by MLDL. *n* = 6–7/group. *p < 0.05, **p < 0.01 vs. sham or MLDL + sham and ^#^*p* < 0.05 vs. I/R. One-way ANOVA was used.

We next investigated whether the mesenteric lymphatic system plays a role in mediating gut I/R-induced lung inflammation. Consistent with published findings (22), lung neutrophil infiltration was significantly increased at 4 h after gut I/R, compared to sham animals (Figure 2A); this increase in neutrophil numbers was significantly inhibited by MLDL (Figure 2A), indicating that the mesenteric lymphatics are involved in lung neutrophil infiltration induced by gut I/R. Likewise, I/R significantly elevated lung MPO levels, an effect attenuated by MLDL (Figure 2B). Trans-endothelial migration of neutrophils involves the opening of endothelial barrier, causing increased permeability (2). Lung microvascular permeability, evaluated by Evans blue extravasation, was markedly elevated after gut I/R, and this effect was ameliorated by MLDL (Figure 2C).

### Post-I/R Lymph, but Not Pre-I/R Lymph, Increases Neutrophil Surface CD11b Expression and Trans-Endothelial Migration

To elucidate whether lymph directly affects endothelial barrier function, we measured TEER as an indicator of endothelial barrier integrity. As a positive control, thrombin (10 U/mL) dramatically reduced TEER, with the most changes at 1 h after treatment (Supplementary Figure 1). In contrast, both pre-I/R (normal) and post-I/R lymph did not significantly alter TEER within 4 h treatment. This indicates that the *in vivo* increase in lung microvascular permeability after intestinal I/R might be due to indirect roles of post-I/R lymph, likely mediated by neutrophils.

**FIGURE 3:**
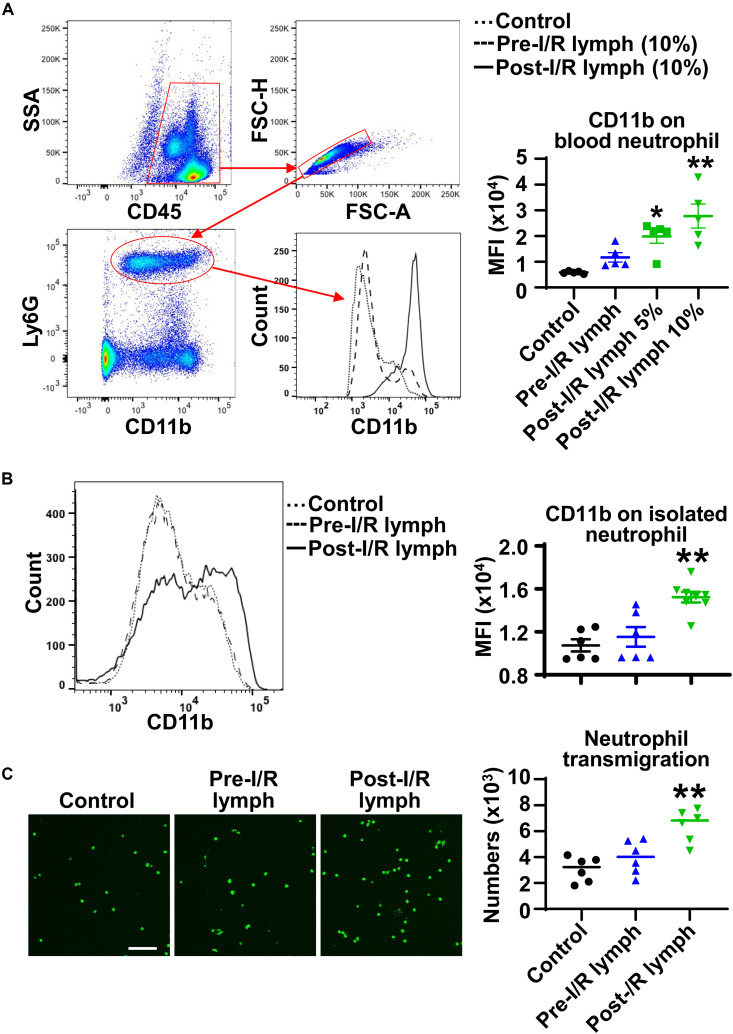
Post-I/R lymph upregulates neutrophil surface CD11b expression and facilitates neutrophil trans-endothelial migration. **(A,B)** Post-I/R lymph, but not pre-I/R lymph, upregulates surface CD11b expression on blood neutrophils **(A)** and on bone marrow derived neutrophils **(B)**. *n* = 5–8/group. **(C)** Post-I/R lymph, but not pre-I/R lymph, promotes neutrophil trans-endothelial migration induced by recombinant CXCL1 (100 ng/mL). *n* = 6/group. *p < 0.05, **p < 0.01 vs. control. One-way ANOVA was used.

During the process of neutrophil diapedesis, CD11b on neutrophil surface is upregulated and binds to endothelial adhesion molecules to form firm adhesion (2). We treated normal mouse whole blood with pre-I/R or post-I/R lymph, and measured neutrophil CD11b expression using multicolor flow cytometry. The data demonstrated that pre-I/R lymph had negligible effect on neutrophil CD11b; in contrast, post-I/R lymph significantly upregulated neutrophil CD11b expression (Figure 3A). To investigate whether post-I/R lymph directly activates neutrophils, we treated isolated bone marrow neutrophils with pre-I/R or post-I/R lymph. Consistently, post-I/R lymph, but not pre-I/R lymph, increased neutrophil surface CD11b expression (Figure 3B). In addition, post-I/R lymph increased neutrophil trans-endothelial migration induced by neutrophil chemoattractant CXCL1 (Figure 3C), consistent with *in vivo* findings showing that blocking mesenteric lymph flow attenuated gut I/R-induced lung neutrophil infiltration.

### Post-I/R Lymph, but Not Pre-I/R Lymph, Stimulates Neutrophil Production of Pro-inflammatory Factors

**FIGURE 4:**
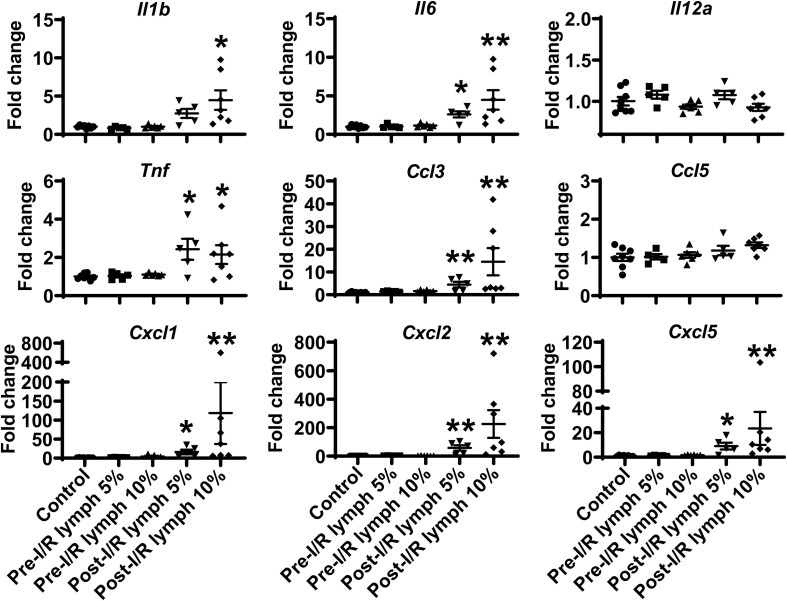
Post-I/R lymph treatment significantly upregulates the expression of pro-inflammatory cytokines and chemokines by neutrophils. Out of 9 mediators measured, 7 showed upregulation (*Il1b*, *Il6*, *Tnf*, *Ccl3*, *Cxcl1*, *Cxcl2*, and *Cxcl5*), and 2 displayed no changes (*Il12a* and *Ccl5*) in post-I/R lymph treated neutrophils. *n* = 5–9/group. *p < 0.05, **p < 0.01 vs. control. One-way ANOVA was used.

The neutrophil exerts their functions through multiple mechanisms, such as respiratory burst, neutrophil extracellular traps formation, degranulation, as well as secretion of pro-inflammatory factors (2). Next, we evaluated the direct effect of lymph on neutrophil functions. Interestingly, our data demonstrated that post-I/R lymph did not directly affect neutrophil respiratory burst, neutrophil extracellular traps formation, and MPO degranulation (Supplementary Figure 2); rather, it significantly upregulated the expression of all pro-inflammatory factors examined with the exception of *Il12a and Ccl5* (Figure 4), indicative of neutrophil activation by post-I/R lymph; on the contrary, pre-I/R lymph had minimal impact on these markers.

### Mesenteric Lymph Is Free of Bacteria, and Post-I/R Lymph Activates Neutrophils Independently of TLR4 Signaling

**FIGURE 5:**
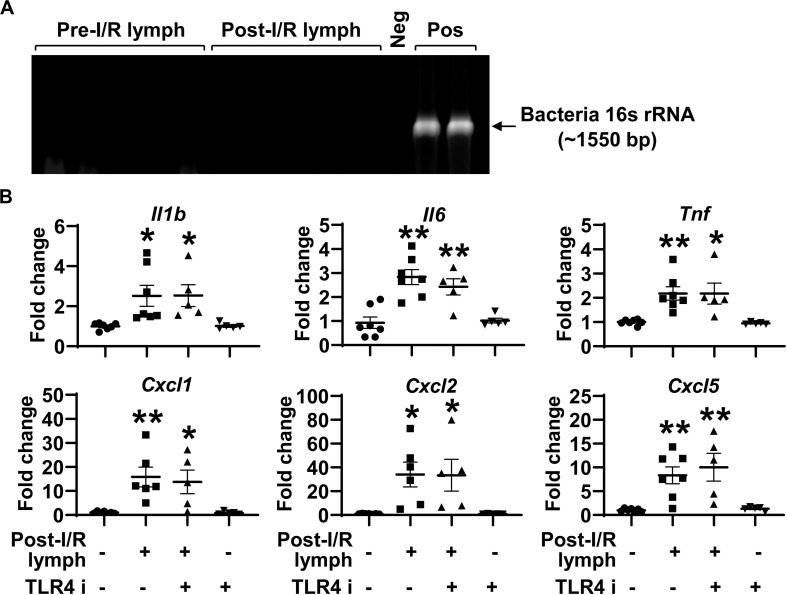
Toll like receptor (TLR) 4 does not mediate post-I/R lymph-induced neutrophil activation. **(A)** Lymph samples, regardless of I/R status, are free of bacteria 16s rRNA. Neg, sterile water. Pos, *pseudomonas aeruginosa*. *n* = 5/group. **(B)** Blocking TLR4 does not affect post-I/R lymph-induced neutrophil upregulation of cytokines and chemokines. TLR4 i, TLR4 inhibitor (1 μM). *n* = 5–7/group. *p < 0.05, **p < 0.01 vs. control. One-way ANOVA was used.

Intestinal I/R impairs epithelial barrier, which allows gut luminal bacteria to spread systemically through mesenteric lymphatics. The activation of TLR4, the receptor for bacteria components (e.g., lipopolysaccharide), induces the generation of pro-inflammatory mediators (21). This raises the possibility that post-I/R lymph can activate TLR4 to increase neutrophil expression of pro-inflammatory mediators. To test this hypothesis, we cultured aliquots of pre-I/R and post-I/R lymph and detected bacteria 16s rRNA using PCR. All lymph samples, regardless of I/R status, were negative for bacteria growth and free of 16s rRNA (Figure 5A), indicating that post-I/R lymph is sterile. Moreover, blocking TLR4 activity did not affect neutrophil activation induced by post-I/R lymph (Figure 5B). These data indicate that bacteria and TLR4 signaling are not involved in post-I/R lymph-induced neutrophil activation.

### Factors > 100 kDa in Post-I/R Lymph Activate Neutrophils

**FIGURE 6:**
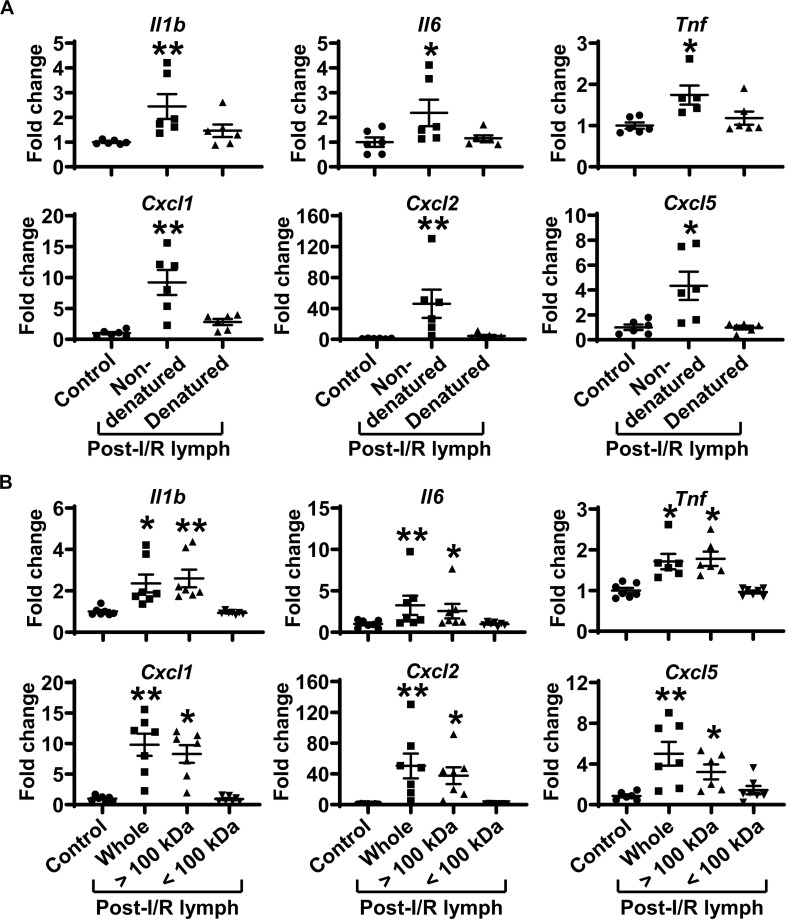
Factors more than 100 kDa in post-I/R lymph activate neutrophils. **(A)** Heat inactivation almost completely abolishes the pro-inflammatory characteristics of post-I/R lymph. **(B)** Factors > 100 kDa in post-I/R lymph stimulate neutrophil generation of inflammatory mediators. *n* = 6–7/group. *p < 0.05, **p < 0.01 vs. control. One-way ANOVA was used.

We denatured post-I/R lymph by heating at 56°C for 30 min to test how this will affect its activity. Heating inactivation almost completely abrogated the pro-inflammatory role of post-I/R lymph (Figure 6A). We then used Amicon Ultra-0.5 Centrifugal Filter Devices to separate lymph into two fractions, based on the protein molecular weight threshold of 100 kDa. The data revealed that while lymph fraction of <100 kDa displayed minimal effect on neutrophil activation, lymph fraction of >100 kDa drastically upregulated neutrophil expression of pro-inflammatory factors to similar levels induced by whole post-I/R lymph (Figure 6B). Therefore, factors >100 kDa in post-I/R lymph are responsible for its neutrophil-activating roles.

Cytokines are known to be able to activate neutrophils. Our data demonstrated that pre-I/R and post-I/R lymph displayed similar levels of interferon-γ, interleukin (IL)-1α, IL-1β, IL-6, IL-17A, IL-22, and tumor necrosis factor (TNF)-α (Supplementary Figure 3). Thus, it is unlikely that these cytokines (molecular weight is ∼15–30 kDa) mediate neutrophil activation elicited by post-I/R lymph, consistent with above data revealing that lymph fraction of <100 kDa had no impact on neutrophil activation.

### Blocking Complement C3a/C5a Receptor or Diamine Oxidase (DAO) Activity Does Not Affect Post-I/R Lymph-Induced Neutrophil Activation

Accordingly, we tested several candidates that are >100 kDa, including complement C3 and DAO. Complement C3 is converted to C3a and C3b. C3b complex cleaves C5 into C5a and C5b. Complement C3a and C5a are potent activators of neutrophils (23). DAO, abundantly expressed in intestinal mucosa, catalyzes the oxidative breakdown of polyamines and histamines. Blood DAO levels are elevated after intestinal I/R (24). We blocked C3a receptor, C5 receptor, or DAO activity using a specific pharmacological inhibitor respectively and examined how this will affect post-I/R lymph-induced neutrophil activation. The results showed that blocking C3a receptor, C5a receptor, or DAO activity had negligible effect on post-I/R lymph elicited neutrophil activation (Supplementary Figure 4). Therefore, other unknown factors, but not complement C3 and DAO, in post-I/R lymph induced neutrophil production of pro-inflammatory mediators.

### NF-κB Pathway Is Responsible for Post-I/R Lymph-Induced Neutrophil Generation of Cytokines and Chemokines

**FIGURE 7:**
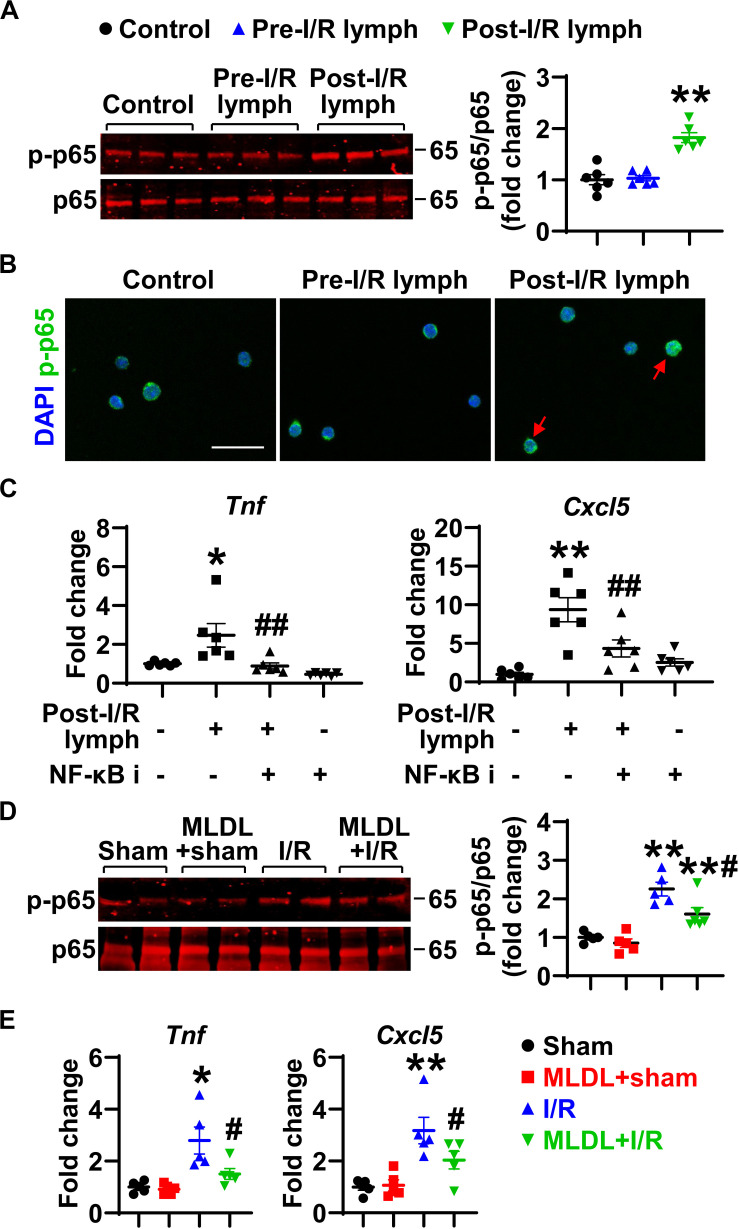
Nuclear factor (NF)-κB partially mediates neutrophil activation induced by post-I/R lymph. **(A)** Post-I/R lymph, but not pre-I/R lymph, activates NF-κB. *n* = 6/group. **(B)** Post-I/R lymph induces NF-κB nuclear translocation. Red arrows indicate nuclear phospho (p)-p65. Representative images are from 3 independent experiments. Scale bar, 20 μm. **(C)** Blocking NF-κB activity attenuates neutrophil *Tnf* and *Cxcl5* gene expression induced by post-I/R lymph. NF-κB i, NF-κB inhibitor (1 μM). *n* = 6/group. *p < 0.05, **p < 0.01 vs. control and ^##^p < 0.01 vs. post-I/R lymph. **(D)** Gut I/R induces neutrophil NF-κB activation, which is inhibited by MLDL. *n* = 5–6/group. **(E)** Gut I/R upregulates the expression of *Tnf* and *Cxcl5* by lung neutrophils, which is suppressed by MLDL. *p < 0.05, **p < 0.01 vs. sham or MLDL + sham and ^#^*p* < 0.05 vs. I/R. One-way ANOVA was used.

The activation of transcription factor NF-κB induces pro-inflammatory gene transcription. We thus evaluated the influence of post-I/R lymph on NF-κB activation. Treatment of neutrophils with post-I/R lymph significantly resulted in p-p65 upregulation and nuclear translocation, indicative of NF-κB activation (Figures 7A,B); by contrast, pre-I/R lymph displayed minimal role on NF-κB activation. Interestingly, inhibition of NF-κB activation significantly attenuated neutrophil expression of *Tnf* and *Cxcl5* induced by post-I/R lymph (Figure 7C), without affecting other inflammatory factors (Supplementary Figure 5). Therefore, these data suggest that post-I/R lymph can activate NF-κB to induce inflammatory mediator generation.

To determine whether the above *in vitro* changes occur *in vivo*, we evaluated NF-κB activation and *Tnf* and *Cxcl5* expression in neutrophils directly from mouse lungs following I/R. The data revealed that gut I/R caused lung neutrophil NF-κB activation, which was attenuated when mesenteric lymph flow was blocked (Figure 7D). Consistent with higher NF-κB activation, neutrophils from I/R mice showed enhanced expression of *Tnf* and *Cxcl5* (Figure 7E). By contrast, neutrophils from MLDL + I/R mice had lower *Tnf* and *Cxcl5* levels than that of I/R animals (Figure 7E). These data further confirmed the role of post-I/R lymph in mediating lung neutrophil activation through the NF-κB pathway.

## Discussion

This study investigated the role of mesenteric lymphatic circulation in lung neutrophil infiltration and activation induced by intestinal I/R. The findings of our study include: (1) during intestinal I/R, blocking mesenteric lymph flow did not alter neutrophil infiltration in local gut tissues but reduced their activity in the lungs and attenuated pulmonary microvascular hyperpermeability; (2) post-I/R lymph, but not pre-I/R (normal) lymph, directly activated neutrophils by upregulating CD11b expression, promoting trans-endothelial migration, and generating pro-inflammatory cytokines and chemokines; (3) the effects of post-I/R lymph to upregulate cytokine production from neutrophils involves NF-κB signaling. Collectively, these findings provide novel insights into the roles and mechanisms of mesenteric lymph-mediated neutrophil activation and ALI following gut I/R.

Although the gut was originally thought to only function in nutrient absorption, it has now been recognized as an immune organ playing a major role in inflammatory responses to injury. Regardless of the type of injury (e.g., trauma, shock, or infection), critically ill patients share one common feature-intestinal hypoperfusion (1, 25). Intestinal ischemia with subsequent reperfusion leads to mucosal injury, barrier failure, and acute inflammatory response. The translocation of gut bacteria and/or pro-inflammatory factors further exacerbates systemic inflammation, ALI, and acute respiratory distress syndrome, if uncontrolled. The gut has two draining systems: portal vein system and mesenteric lymphatics (5, 26). While the portal vein was historically considered the major conduit, a body of evidence shows that the mesenteric lymphatics serve as an equally important route in delivery of injurious factors to remote organs (10, 27). This is consistent with our results demonstrating that blocking mesenteric lymph duct ameliorated lung neutrophil infiltration and microvascular permeability after gut I/R. The attenuated lung inflammation in mesenteric lymph duct ligation (MLDL) + I/R mice is not through improving gut injury because I/R and MLDL + I/R mice had similar levels of gut neutrophils; rather, the mesenteric lymph carries pro-inflammatory factors that cause neutrophil activation and ALI.

ALI is characterized by endothelial barrier dysfunction and neutrophil infiltration. Studies have shown that *in vitro* post-hemorrhagic shock lymph can induce endothelial permeability and death after 18 h treatment (28). However, our data indicated the direct effect of post-I/R lymph on endothelial barrier function is rather minor, at least for the time at 4 h treatment. We focused on this time point because our *in vivo* results displayed increased permeability at 4 h after I/R. Therefore, the elevated lung endothelial permeability after gut I/R is not likely due to a direct effect of post-I/R lymph on endothelial cells. In contrast, post-I/R lymph exerts a direct effect on neutrophil activation, as treatment of neutrophils with post-I/R lymph, but not pre-I/R lymph, upregulates CD11b surface expression, promotes neutrophil trans-endothelial migration, and upregulates pro-inflammatory mediators. Supporting this is the *in vivo* finding that in the absence of mesenteric lymph return, I/R-induced neutrophil activities in the lungs were greatly reduced.

Our data demonstrated that post-I/R lymph is capable of stimulating neutrophils to produce a wide range of pro-inflammatory cytokines, including IL-6 and TNF-α. In contrast to the previous findings of increased IL-6 and TNF-α in post-I/R lymph (29, 30), our data showed comparable levels of IL-6 and TNF-α in pre-I/R and post-I/R lymph, arguing against the likelihood that the observed increases in neutrophil activity result from an increased level of these molecules in the gut lymph. The discrepant results in lymph IL-6 and TNF-α levels might be partly explained by the difference in lymph collection place. We collected lymph directly from mesenteric lymph duct, while the above studies collected lymph via thoracic duct (29, 30), which drains around 75% of the lymph from the entire body.

In an effort to characterize the molecular identify of pro-inflammatory factors in post-I/R lymph, we found them to concentrate in the fraction of larger than 100 kDa. This is consistent with a published report demonstrating that molecules larger than 100 kDa in post-hemorrhagic shock lymph are biologically active (28). Our initial hypothesis was focused on two potential candidates that are greater than 100 kDa: complement C3 and DAO, based on previous proteomics analysis demonstrating that complement C3 is upregulated in post-shock mesenteric lymph (31). C3a and C5a are known to be involved in tissue I/R injury (32), and C5a inhibitor protects against small intestine I/R injury (33). There is also strong literature support for the effect of I/R to damage gut villi and release cytoplasmic DAO, causing increased blood level and decreased gut activity of DAO (24). However, our data demonstrated that blocking C3a, C5a, or DAO did not affect the pro-inflammatory effects of post-I/R lymph, suggesting that these factors do not play a major role in post-I/R lymph-induced neutrophil activation.

TLR4 acts as sensors of microbial infection and tissue injury by recognizing pathogen associated molecular patterns and damage associated molecular patterns respectively (34, 35). Injection of post-hemorrhagic shock lymph into naïve wild type mice caused lung injury, which was fully prevented in TLR4^–/–^ mice (36). This indicates that there may exist TLR4 ligands in the post-injury lymph that can activate TLR4 signaling to induce ALI. Our data showed that post-I/R lymph did not contain bacteria, excluding the existence of bacteria-derived TLR4 ligands in the lymph, consistent with other reports that mesenteric lymph after hemorrhagic shock or burn injury is sterile (28, 37). Furthermore, blocking TLR4 did not affect neutrophil activation induced by post-I/R lymph, indicating that TLR4 signaling may play a minimal role in sterile inflammatory response to I/R injury in the gut lymph system. The differences between hemorrhagic shock vs. I/R may be attributable to the discrepant results of our studies and others. First, I/R induces gut local injury initially, while hemorrhagic shock triggers disseminated and systemic alterations of homeostasis. Moreover, our model is based on 30-min gut ischemia, while 60-min severe hemodynamic disturbance (shock) was induced in the above study (36).

The NF-κB pathway is a typical inflammatory signaling pathway; its activation induces the expression of proinflammatory genes including cytokines, chemokines, and adhesion molecules (38). Our data showed that post-I/R lymph activated NF-κB, and blocking NF-κB activation attenuated *Tnf* and *Cxcl5* gene expression induced by post-I/R lymph. The involvement of NF-kB signaling is further supported by our *in vivo* observation that blocking mesenteric lymph flow attenuated lung neutrophil NF-κB activation and generation of *Tnf* and *Cxcl5*. TNF-α is known to prime neutrophils for adhesion and transmigration (39), and CXCL5 is a potent chemoattractant for neutrophils (40). This may form a positive feedback cycle for lung neutrophil infiltration and activation following gut I/R.

**FIGURE 8:**
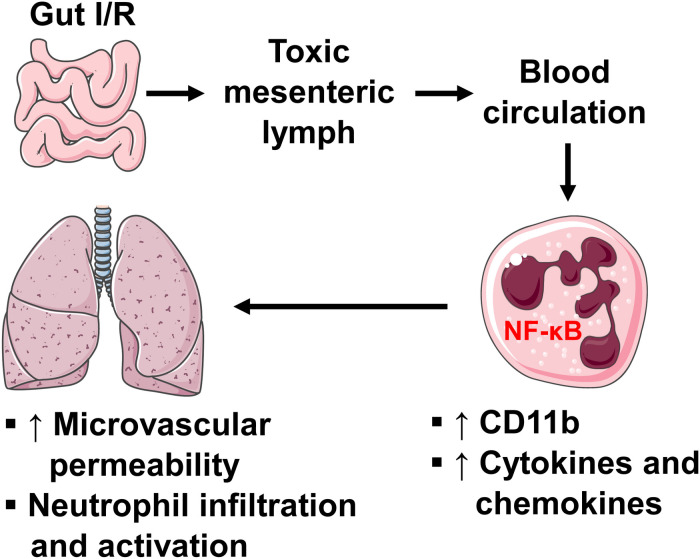
A diagram showing how intestinal I/R induces ALI. Intestinal I/R induces mucosal injury and gut barrier disruption, causing the release of injurious factors. The mesenteric lymph carries those harmful factors and drains to blood circulation, where lymph upregulates neutrophil surface CD11b and production of pro-inflammatory factors through a mechanism involving NF-κB, leading to pulmonary microvascular hyperpermeability as well as neutrophil infiltration and activation.

In summary, our study reveals the role of intestinal lymphatics in bridging gut I/R injury and remote lung injury through activating neutrophils. We propose that during gut I/R injury, local tissue inflammation causes the release of bioactive agents into the mesenteric lymph, followed by dissemination in the systemic and pulmonary blood circulation, where lymph activates neutrophils and induces lung injury via a mechanism involving NF-κB signaling (Figure 8). Although the exact molecular characteristics of the lymph factors remain unclear, proteins of large size (>100 kDa) appear to be responsible for activating neutrophils during gut I/R. Future studies that utilize proteomics, lipidomics, and/or nanotechnology may facilitate the identification of these culprit factors.

## Data Availability Statement

All datasets presented in this study are included in the article/Supplementary Material.

## Ethics Statement

The animal study was reviewed and approved by Institutional Animal Care and Use Committee at the University of South Florida.

## Author Contributions

YM performed, analyzed, and interpreted most of the experiments. AC carried out PCR analysis of bacteria in lymph samples. TZ assisted with histology and immunoblotting experiments. XY, VC, NV, EBK, and MHW participated in experimental design and data interpretation. SYY conceived the idea and directed the project throughout all levels of development. All the authors discussed the results and approved the manuscript.

## Conflict of Interest

The authors declare that the research was conducted in the absence of any commercial or financial relationships that could be construed as a potential conflict of interest.

## Abbreviations

ALI: acute lung injury
DAO: diamine oxidase
IL: interleukin
I/R: ischemia/reperfusion
MFI: mean fluorescent intensity
MLDL: mesenteric lymph duct ligation
MPO: myeloperoxidase
NF-κ B: nuclear factor- κ B
PMA: phorbol myristate acetate
p-p 65: phospho-p65
TEER: transendothelial electrical resistance
TLR4: toll like receptor 4
TNF: tumor necrosis factor.
