# Supplementary material for: Gut Ischemia Reperfusion Injury Induces Lung Inflammation via Mesenteric Lymph-Mediated Neutrophil Activation
Source: Front Immunol. 2020 Sep 11;11:586685. doi: 10.3389/fimmu.2020.586685 (PMC7517702; doi:10.3389/fimmu.2020.586685)
Supplement: Supplementary file 1 [file Data_Sheet_1.docx]

***Supplemental Material***

**
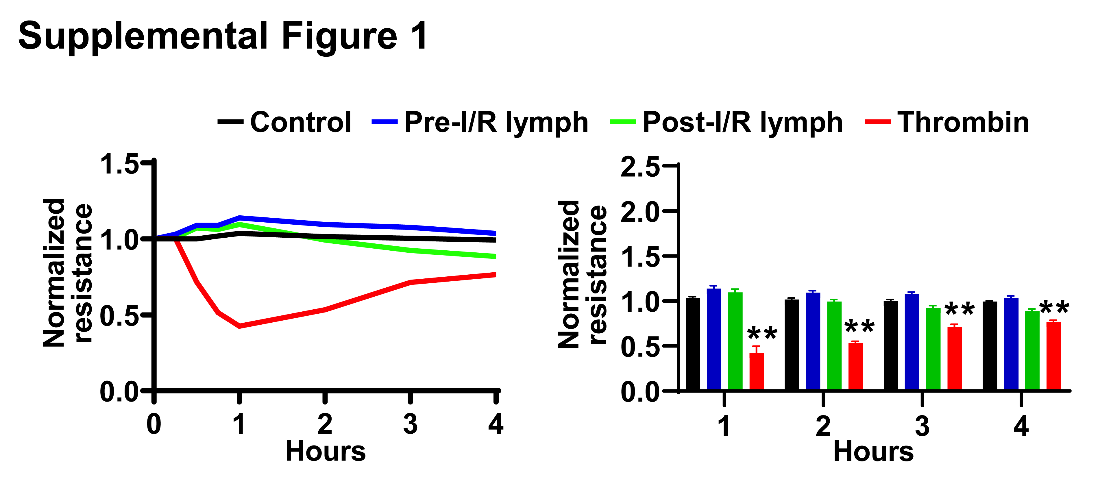
**

**Supplemental Figure 1. Pre-I/R or post-I/R lymph does not affect endothelial barrier integrity.** Pre-I/R and post-I/R lymph does not significantly alter trans-endothelial electrical resistance (TEER) within 4 h treatment. Thrombin (10 U/mL), as a positive control, reduces TEER. n=8-12/group. **p<0.01 vs. control. One-way ANOVA was used.

**
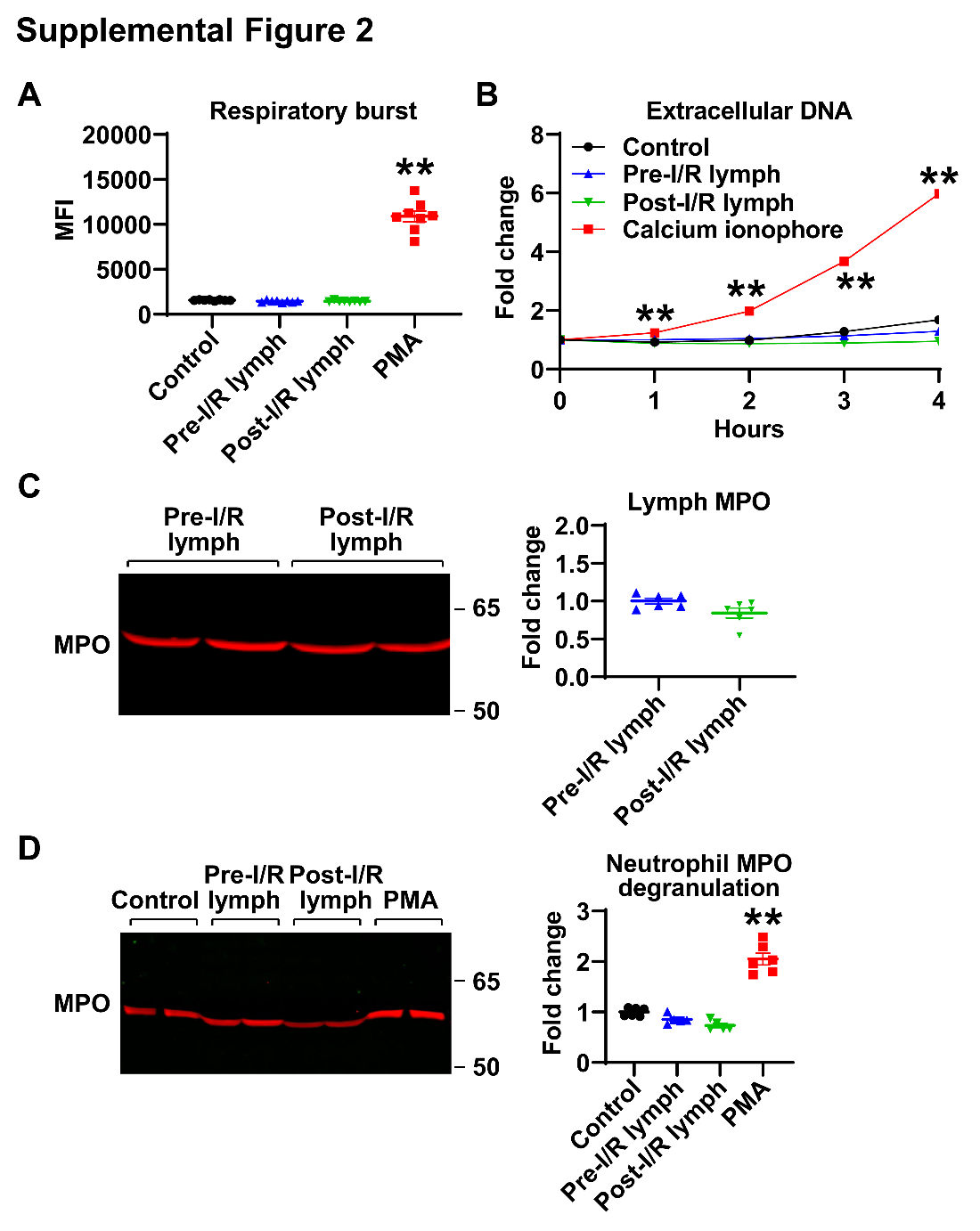
**

**Supplemental Figure 2. Post-I/R lymph does not affect neutrophil respiratory burst, neutrophil extracellular traps formation, and MPO degranulation. (A)** Pre-I/R and post-I/R lymph exhibit no effect on neutrophil respiratory burst. Phorbol myristate acetate (PMA, 200 nM), as a positive control, induces neutrophil respiratory burst. n=8/group. **(B)** Pre-I/R and post-I/R lymph display no impact on the release of extracellular DNA by neutrophils, an indicator of neutrophil extracellular traps formation. Calcium ionophore (5 μM), as a positive control, induces extracellular DNA release. n=8-9/group. One-way ANOVA was used. **(C)** Pre-I/R and post-I/R lymph show comparable MPO levels. n=6/group. T-test was used. **(D)** Pre-I/R and post-I/R lymph have no impact on neutrophil degranulation of MPO. PMA (200 nM), as a positive control, significantly induces MPO degranulation. n=5-6/group. **p<0.01 vs. control. One-way ANOVA was used.

**
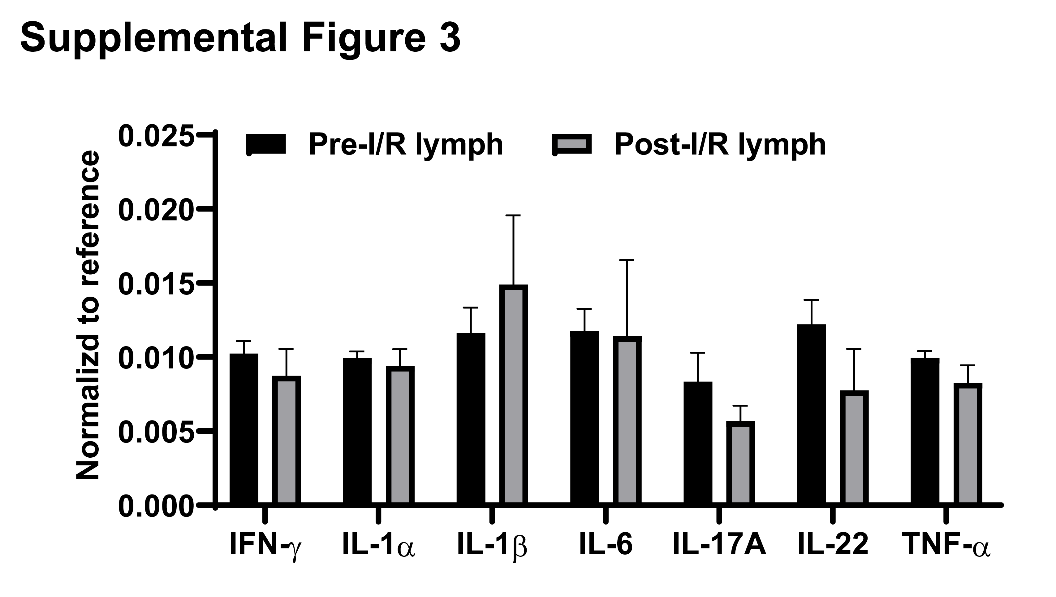
**

**Supplemental Figure 3. Pre-I/R and post-I/R lymph show comparable levels of cytokines.** Cytokine levels in the lymph were semi-quantitated using a rat XL cytokine array kit. n=4/group. T-test was used.


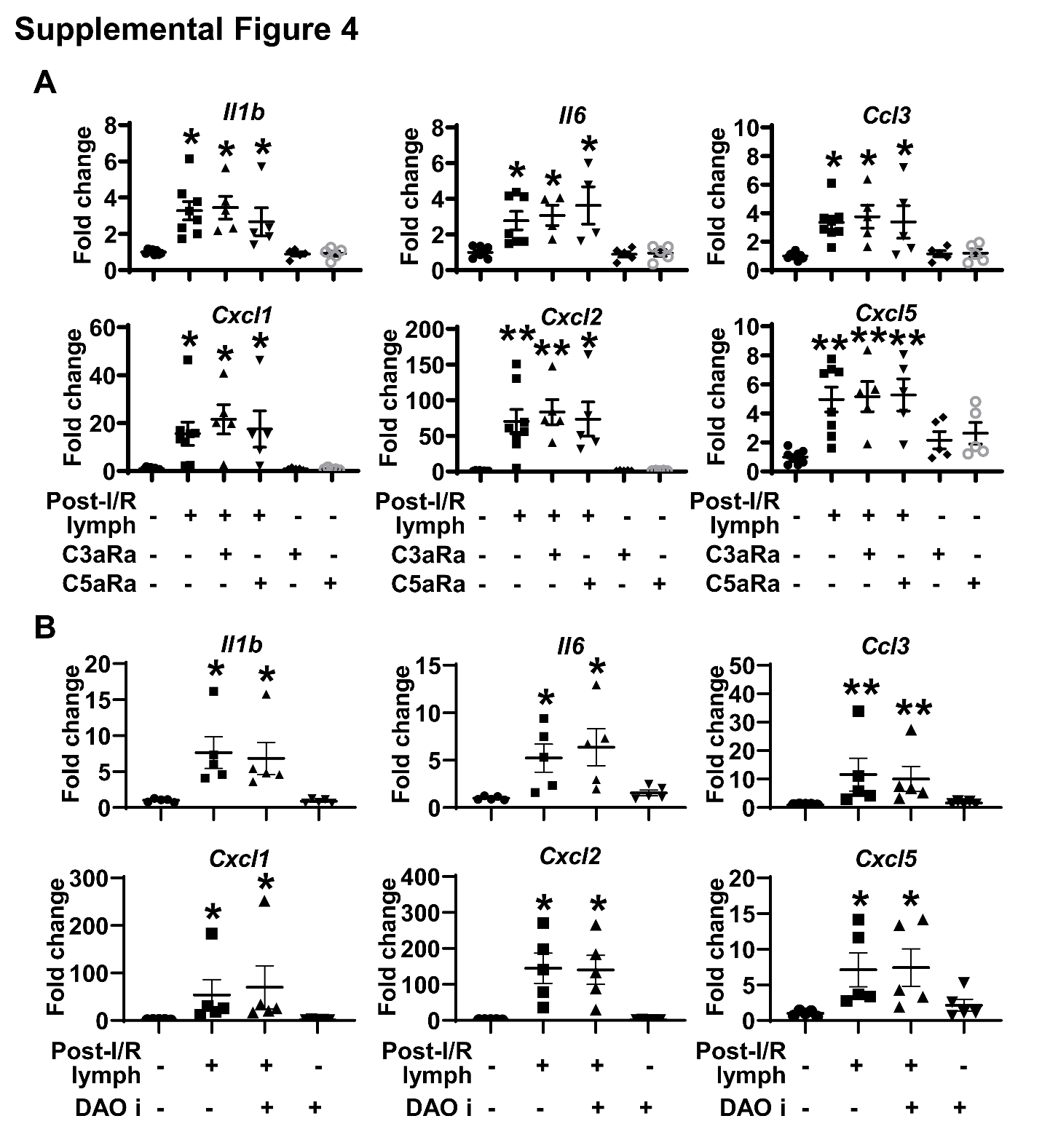


**Supplemental Figure 4. Blocking complement C3a receptor, C5a receptor, or diamine oxidase (DAO) activity does not affect post-I/R lymph-induced neutrophil activation.** C3aRa, C3a receptor antagonist (1 μM); C5aRa, C5a receptor antagonist (1 μM); DAO i, DAO inhibitor (1 mM). n=5-8/group. *p<0.05, **p<0.01 vs. control. One-way ANOVA was used.


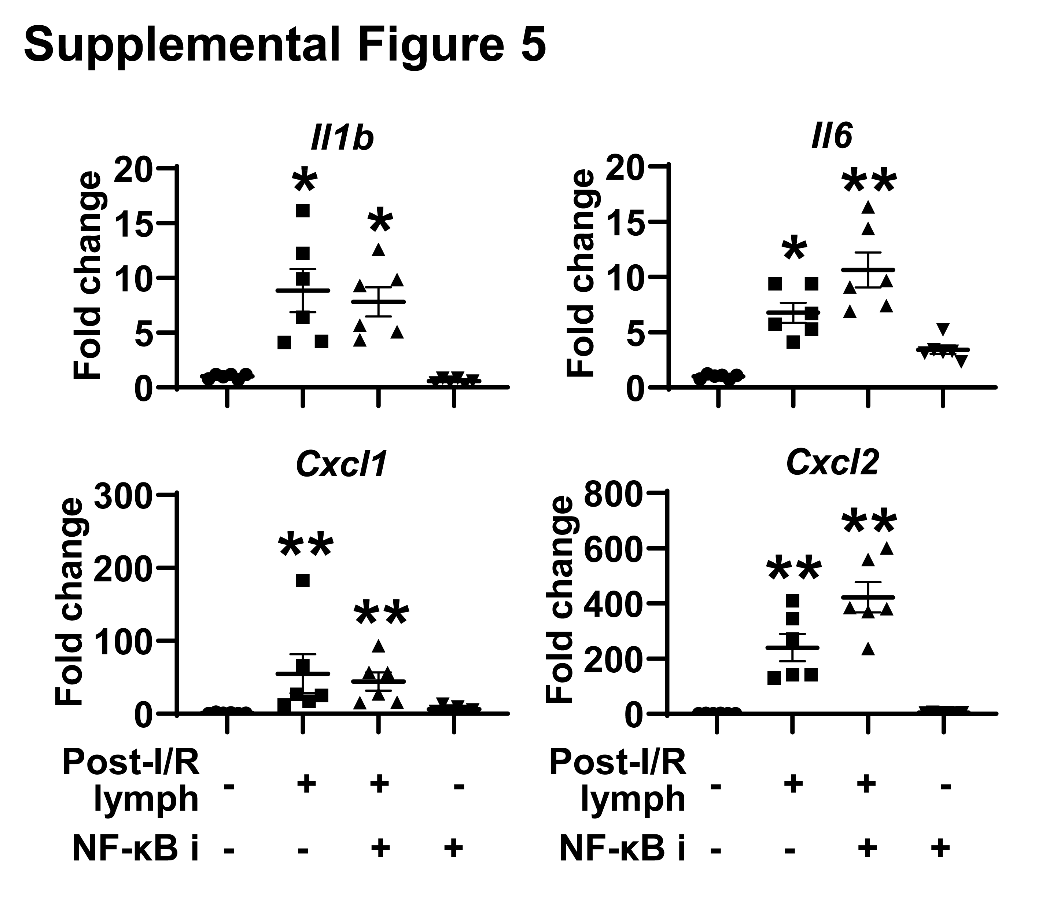


**Supplemental Figure 5. Blocking NF-κB activity does not affect neutrophil production of *Il1b*, *Il6*, *Cxcl1*, and *Cxcl2* induced by post-I/R lymph.** n=6/group. *p<0.05, **p<0.01 vs. control. One-way ANOVA was used.
